# Supplementary Surveillance of Poliovirus Circulation in the Russian Federation: Results of a Study on Migrant Children of “Risk Group”

**DOI:** 10.3390/v17060746

**Published:** 2025-05-23

**Authors:** Olga E. Ivanova, Yulia M. Mikhailova, Nadezhda S. Morozova, Alina V. Chirova, Evgeniya A. Cherepanova, Lyudmila N. Golitsyna, Olga Y. Baikova, Elizaveta V. Yakovchuk, Evgenia V. Karpova, Liubov I. Kozlovskaya

**Affiliations:** 1 Federal State Autonomous Scientific Institution “Chumakov Federal Center for Research and Development of Immune-and-Biological Products of the Russian Academy of Sciences” (Institute of Poliomyelitis) (FSASI “Chumakov FSC R&D IBP RAS”), 108819 Moscow, Russia; baykovaaa@mail.ru (O.Y.B.); yakovchuklisa@gmail.com (E.V.Y.); evg_karpova@mail.ru (E.V.K.); 2 Department of Organization and Technology of Production of Immunobiological Preparations, Institute for Translational Medicine and Biotechnology, First Moscow State Medical University (Sechenov University), 119048 Moscow, Russia; 3 Federal Budgetary Health Institution “Federal Center of Hygiene and Epidemiology” of the Federal Office for Inspectorate in the Field of Customers and Human Well-Being Protection, 117105 Moscow, Russia; mikhailovaym@fcgie.ru (Y.M.M.); morozovans@fcgie.ru (N.S.M.); chirovaav@fcgie.ru (A.V.C.); cherepanovaea@fcgie.ru (E.A.C.); 4 Academician I.N. Blokhina Nizhny Novgorod Scientific Research Institute of Epidemiology and Microbiology, 603950 Nizhny Novgorod, Russia; lyudmila_galitzina@mail.ru

**Keywords:** poliomyelitis, vaccine-derived poliovirus, VDPV2, non-polio enteroviruses, migrants

## Abstract

The detection of “silent” poliovirus (PV) circulation among clinically healthy populations is an important component of supplementary surveillance for poliomyelitis. Migrants from countries or regions where polio is endemic, affected by outbreaks, or at risk may contribute to the introduction of PVs of epidemic significance: wild poliovirus type 1, vaccine-derived polioviruses (VDPVs), or poliovirus type 2 into polio-free countries. Migrant children, refugees under 5 years of age, are considered a “risk group” in Russia and are subject to testing for PVs. During 2014–2023, guided by the algorithm of virological and molecular investigation of acute flaccid paralysis cases recommended by the WHO, 51,548 migrant children, arriving from 40 countries, were examined. Among 4% of children excreting various cytopathogenic viruses, polio excretors accounted for 20.8%. Among the PVs, PV3 was predominant (41.7%), and PV types 2, 1, and a mixture of PVs accounted for, respectively, 28.2%, 18.8%, and 11.3%. All isolates of PVs 1 and 3 were identified as Sabin-like. The detection of five children excreting epidemically significant PV2 (four VDPV2 and one Sabin-like) required an assessment of the risk of dissemination and additional immunization activities. Among 580 identified isolates of NPEV, the most abundant was the *E. betacoxsakie* species at 73.8% (CVB1–6, E11, E6, E13, E7). Information on NPEVs expands our knowledge of the spectrum of NPEVs circulating among healthy children worldwide, but its prognostic significance is still unclear. The detection of PVs in children from the “risk group” allows targeted anti-epidemic measures and is a significant advantage of this type of supplementary surveillance for polio.

## 1. Introduction

A feature of poliovirus infection, caused by an RNA-containing virus of the genus *Enterovirus* of the family *Picornaviridae*, is that the paralytic form of poliomyelitis develops in approximately 1% of those infected [1]. The goal of the World Health Organization (WHO) Global Polio Eradication Initiative (GPEI) is to eliminate the existence of wild (w) poliovirus (PV) and vaccine-derived PV (VDPV) in nature, so the detection of “silent” PV circulation among clinically healthy individuals is an important part of polio surveillance. Certain populations (“risk groups”), among whom the circulation of PV is most likely, are of great importance. People belonging to the “risk group” depends on the specific epidemiological situation regarding polio in the world, or in a particular country or region.

Since 2014, the WHO has considered the international spread of wPVs a “public health emergency of international concern (PHEIC)” [2]. Despite the outstanding progress in the field of combating polio, this assessment of the current situation remains to this day. The possibility of the spread of epidemiologically significant PVs (wPV type 1, VDPV, PV2), not only between countries sharing common borders but also between different continents, has been clearly demonstrated in recent years. WPV type 1, circulating in Pakistan in 2019, caused polio outbreaks in Malawi and Mozambique in 2021 [3]. Genetically related isolates of circulating VDPV type 2 (cVDPV2) were detected in Israel, the United States, the United Kingdom, and Canada in 2021 [4,5,6,7,8]. Analysis of genetically related cVDPV2 isolates detected in 2024 in wastewater samples in Finland, Germany, Poland, Spain, and the United Kingdom allowed the conclusion that they were imported almost simultaneously from an unknown country and possibly circulated within European countries [9]. A significant contribution to the spread of PVs can be made by marginalized groups of the population (refugees, illegal migrants, nomadic populations) and labor migrants from polio-endemic or polio-risk countries. European countries, a polio-free region since 2002 [10], have been accepting the largest number of representatives of these groups in the last decade—approximately 36% of the world’s number of international migrants, 13% of the total population of the European region. There are 101 million migrants living in Europe, including 12.5 million refugees [11]. Recognizing that migration is a part of society, the WHO Regional Office for Europe considers that, overall, the risk of transmission of infectious diseases from refugees and migrants to host populations is low [11]. However, migration typically occurs from countries with limited or disrupted healthcare and vaccination systems, creating a risk of pathogen spread and outbreaks of vaccine-preventable diseases. Between 2000 and 2020, 47 outbreaks of vaccine-preventable diseases (mainly measles, varicella, hepatitis A, rubella, mumps) involving migrants occurred in 13 European countries [12]. In 2021, VDPV2, associated with the polio outbreak in Tajikistan, originating from the Pakistan cluster, was isolated in Ukraine from 2 polio cases and 19 healthy contacts [13].

A large number of migrants and their family members enter the Russian Federation every year, including from polio-risk countries. In 2022, 11.6 million migrants (8% of the population) lived in the country [14]. Events of wPV1 importation by migrants from Tajikistan were identified in Russia in 2010; the virus was isolated from both polio patients and healthy individuals [15,16]. Given the continuing risk of importation of epidemiologically significant PVs from polio-endemic countries and countries of VDPV circulation, the Russian national polio surveillance system considers healthy children from migrant families, nomadic populations, and persons arriving from polio-risk countries or internal regions as a target group for PV testing (“risk group”) [17].

Therefore, the aim of the study was a virological examination of clinical materials from children belonging to the migrant “risk group” to identify PVs, assess risks, and subsequently take the necessary anti-epidemic measures.

## 2. Materials and Methods

### 2.1. Organization of Research and Collection of Materials

In the contingent of studied individuals, the procedure of collection and laboratory investigation of materials was performed in accordance with the Russian regulatory, methodological, and organizational guidelines [17,18,19]. The examined group included healthy children under 5 years of age from “risk groups” (migrant families, nomadic population groups, refugees and forced migrants, persons arriving from countries/regions endemic or at risk for polio). The region of the North Caucasus of Russia was considered a region of risk. Children from “risk groups” were identified and subjected to investigation when applying for migration registration, seeking medical care, or visiting social welfare institutions. One stool sample was collected from each child; if wPV, VDPV, or PV2 were detected, the collection was continued until a negative result was obtained.

### 2.2. Algorithm of Laboratory Investigation

The preparation of fecal suspensions and virus isolation on RD, HEp2c (Cincinnatti), and L20B cell lines obtained from WHO-approved sources were performed in accordance with the WHO recommendations [20]. The algorithm for the identification and intratypic differentiation (ITD) of PV changed over time in accordance with the WHO recommendations. The identification of PVs was performed using a neutralization assay with polyclonal sera (RIVM, Bilthoven, The Netherlands) for the identification of poliovirus type 1–3 [20]. In different years, ITD was carried out using an ELISA [20,21], RT-PCR, or real-time RT-PCR [22,23] and an additional set of primers for the identification of new oral poliovirus vaccine nOPV2 derivatives (CDC, Atlanta, GA, USA) [24]. In case of the detection of epidemiologically significant PV, partial genome sequencing was performed [24]. The use of cell cultures allows, along with PVs, the isolation of cytopathogenic non-polio enteroviruses (NPEVs) and adenoviruses (HAdVs). The identification of NPEVs was performed using a neutralization assay according to the standard WHO protocol [20] with pooled polyclonal sera (RIVM, Bilthoven, The Netherlands) to identify 50 NPEV types (pools A–G and pools H-R) and 1 parechovirus. In recent years, NPEV types were identified or confirmed by VP1 genome region sequencing [25]. Adenoviruses were provisionally identified by a typical grape-like cytopathic effect in HEp2c cell cultures and verified by PCR [26].

### 2.3. Ethics of Research

This study was conducted as part of the Polio and Acute Flaccid Paralysis (AFP) Surveillance Program in the Russian Federation, and specialized informed consent was not required. The parents or guardians of the study participants were informed and signed their informed consent for clinical specimen collection and investigation for diagnostic purposes.

## 3. Results

**Figure 1:**
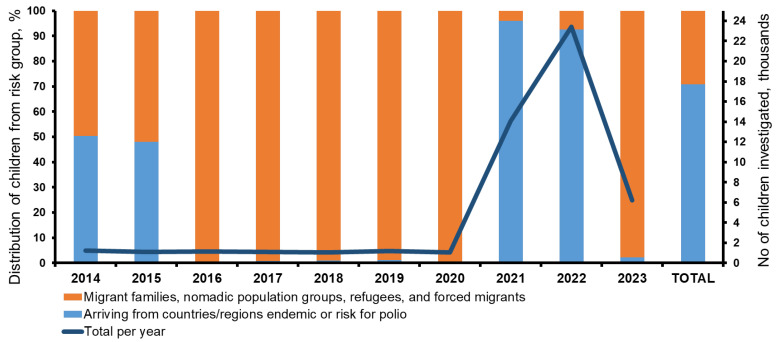
Number and distribution of children in risk groups investigated during the study in 2014–2023. The right vertical axis and graph are the number of children studied; the left vertical axis is the distribution of children by groups, %; the horizontal axis is the year.

Over the course of 10 years (2014–2023), 51,548 children from two risk groups were examined: (1) migrant families, nomadic population groups, refugees, and forced migrants; (2) arriving from countries/regions endemic or at risk for polio (Figure 1). In different years, depending on the geopolitical or epidemiological situation regarding polio in the world, the number of children in the particular groups varied. Until 2016, the number in both groups was approximately the same; since 2016, the number of children from the group of migrants and forced migrants has sharply increased; in 2021–2022, the number of children in the group arriving from polio-risk countries increased, which was associated with a polio outbreak in 2021 in Tajikistan and Ukraine. Overall, the largest number of children tested belonged to this group (36,509, 70.8%). The smallest number of children from risk groups (1067 people) were examined in 2020, due to a significant decrease in migration because of restrictive measures implemented in response to the COVID-19 pandemic.

Risk-group children arrived from 40 different territories, including internal migration from the Russian region of the North Caucasus, and the majority external migrants arrived from Central Asian countries (25,000, 48.5%). Migrants from Tajikistan predominated in this group (22,668 children, 90.7%). Migrants from Ukraine made up 34.6% (17,857 children), and children from the North Caucasus region made up 12.6% (6513 children). Children from other countries, including polio-endemic Afghanistan and Pakistan, and African countries with VDPVs circulation, were represented by a small number of children, ranging from 1 to 70.

**Figure 2:**
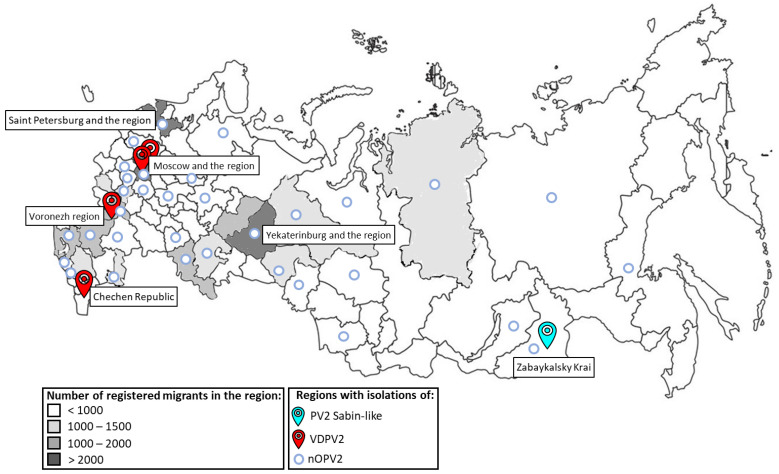
Regions of the Russian Federation where children from migrant risk groups and polioviruses of epidemiological significance were identified in 2014–2023.

Most children were identified in 18 federal subjects (regions) of the country (Figure 2). The largest number of children were identified in Moscow and the Moscow region (8290, 16.1%), which is at least three-times higher than the number of children examined in the other regions (within 1000–2000). This is logical, since the country’s capital is a major industrial center and a key transport hub, through which the main migration flows pass. A significant number of children were identified in two other large cities and transport hubs: St. Petersburg and the region (2012 children, 3.9%), and Yekaterinburg and the region (2087 children, 4.0%).

Overall, cytopathogenic viruses were isolated from 2077 (4%) children (Table 1). NPEVs and HAdVs were isolated from 1645 children (79.2%): 1602 children (97.4%) excreted NPEVs and 43 (2.6%) HAdVs. PVs solely or in mixtures with other viruses were isolated from 432 children (20.8%). Among PVs, PV type 3 dominated (180 children, 41.7%), and PV types 2, 1, and PV mixtures accounted for, respectively, 28.2% (122 children), 18.8% (81 children), 11.3% (49 children).

### 3.1. Polioviruses

All isolates of PV types 1 and 3 were identified as Sabin-like. Following the global cessation of the use of the trivalent oral poliovirus vaccine from Sabin strains (tOPV) in April 2016 (the so-called “switch”) [27], the isolation of any PV2 has been considered of epidemiological significance. Among the seven PV2 isolates detected in 2016, two were detected after the “switch” and identified as VDPV2. The viruses were isolated from two healthy children, unvaccinated against polio (Table 2), one of whom arrived in Moscow from the Chechen Republic in the North Caucasus region, and the other of whom permanently lived there. The children were relatives and were in contact with each other [28].

In 2021, in relation to the cVDPV2 outbreak in Tajikistan and Ukraine, additional measures were implemented in Russia to screen children under 5 years of age arriving from these countries. Since nOPV2 from the genetically modified Sabin strain approved by WHO for emergency use [29], but not registered in Russia, was used to combat the outbreak in Tajikistan, the detection of nOPV2 isolates from vaccinated Tajikistan children was considered an epidemiologically significant event. Overall, PV2 was isolated from 109 children from Tajikistan: 106 isolates were identified as nOPV2 derivatives, and 2 isolates from healthy children were identified as VDPV2, genetically related to the outbreak virus in Tajikistan [24]. The same year, a vaccine-like PV2 was isolated from a 9-month-old healthy child (Table 2) vaccinated against polio arriving from Egypt, where additional immunization measures were carried out in response to the cVDPV2 detection using monovalent OPV2 [30].

### 3.2. Response Measures

In each case of PV2 isolation, an epidemiological investigation and a full range of sanitary and anti-epidemic (preventive) measures were carried out in accordance with the Russian sanitary and epidemiological requirements [17], as well as the WHO standard operating procedure for responding to an event associated with poliovirus [31]. These measures included risk assessment of PV spread, quarantine of virus excretors, investigation of healthy contacts, immunization against polio within the boundaries of the event detection area, strengthening routine polio/AFP surveillance, and intensification of wastewater monitoring. As a part of the response measures, a multi-level analysis of immunization against polio of the population of the municipality and the region as a whole was also conducted, followed by catch-up immunization using IPV. As a part of supplementary immunization, more than 40 thousand children were vaccinated in 2016–2017, and more than 146 thousand in 2021.

Information on VDPV2 isolation events was sent to WHO in accordance with the International Health Regulations [32] and the National Action Plan for Maintaining the Polio-Free Status of the Russian Federation [33].

### 3.3. Non-Polio Enteroviruses (NPEVs)

Over the observation period, 1602 NPEV isolates were obtained from the studied cohort, of which 580 were identified (Table 3). The NPEVs belonged to three species of human NPEVs, and most of the 580 identified isolates belonged to the Enterovirus betacoxsakie species (428 isolates, 73.8%), while viruses of the *E. alphacoxsakie* and *E. coxsackiepol* species included 107 (18.4%) and 45 (7.8%) isolates, respectively. *E. alphacoxsakie* species included mostly CVA4 (45 isolates, 42.1%), CVA10 (20 isolates, 18.7%), and EV-A71 (7 isolates, 6.5%). *E. betacoxsakie* species were represented predominantly by the CVB1–6 group (206 isolates, 48.1%), E11 (52 isolates, 12.1%), E6 (43 isolates, 10.0%), E13 (29 isolates, 6.8%), and E7 (22 isolates, 5.1%). In the CVB1–6 group of 59 identified isolates, the majority were CVB5 (20 isolates, 33.9%), CVB2 (16 isolates, 27.1%), and CVB4 (13 isolates, 22%). The most predominant *E. coxsackiepol* species type were CVA19 and CVA24 (12 isolates each, 26.7%).

## 4. Discussion

The accumulated experience of the GPEI convincingly demonstrates the importance of supplementary types of polio surveillance in the detection of the “silent” circulation of epidemiologically significant PVs. Countries in the WHO European Region combine AFP surveillance with either enterovirus surveillance or environmental surveillance, or, like the Russian Federation, implement all three types of surveillance [34]. Studies of children from “risk groups” are usually carried out irregularly, depending on the epidemiological situation, for example, the arrival of a large migrant group in the country [35]. The composition of the group and the format of the study also do not follow a prescribed algorithm; the most common are serological studies to determine the level of immunity [36,37,38]. In Russia, the criteria for the formation of “risk groups” and the algorithm for investigation are included in regulatory and methodological documents; the number of materials and the national or administrative composition of the group may be changed according to the situation concerning polio. Permanent monitoring of the “risk group” made it possible to detect “silent” epidemiologically significant PVs and immediately carry out the necessary anti-epidemic measures. It should be noted that the virological testing of large groups of people organizationally is a more difficult task than, for example, wastewater monitoring, and the operational and financial burden on the laboratory increases. However, unlike environmental surveillance, the study of “risk groups” allows the precise identification of the viral excretor and the implementation of timely targeted measures to prevent the spread of the virus. This is a significant advantage of this approach, given that asymptomatic virus excretion can continue for quite a long time, facilitating dissemination of the virus in the population and its possible circulation. For example, cVDPV2 excretion by a child from Tajikistan continued for at least 85 days from the moment of crossing the Russian border [24]. It is noteworthy that the PVs isolated from this child in September were more altered compared to the Sabin2 strain than isolates from patients in Tajikistan during the entire outbreak period (November 2020–June 2021), 3.99–4.32% and 2.21–2.88% differences, respectively [24]. In this regard, improvement of the laboratory algorithm for fecal samples investigation is of fundamental importance: the introduction of direct molecular detection of PV in fecal samples will make it possible to radically reduce the analysis time.

An algorithm for laboratory testing of fecal samples (using cell cultures to isolate the virus) made it possible to obtain information about NPEVs excreted by healthy children from “risk groups”. These data expand our knowledge of asymptomatic circulating NPEVs, particularly in countries where surveillance for enterovirus infections is not conducted. However, the prognostic significance of these data and the role of imported NPEVs in the occurrence of non-polio enterovirus infection among residents of the host country are not clear either. Such data are difficult to compare, since monitoring examinations of healthy child-residents for NPEVs are not carried out regularly. A comparison of our data and long-term data on NPEVs isolated in Russia from cases of enteroviral infection and from the environment obtained by the Reference Center for Enterovirus Infection Monitoring in Nizhny Novgorod (https://www.nniiem.ru/development/informanalit/evi.html, accessed on 18 May 2025) showed that the spectrum of NPEV types in different types of surveillance did not coincide. In addition, it cannot be ruled out that the migrant child was infected with NPEV after arriving in the country of destination. This consideration underscores the importance of the timing of clinical specimen collection when designing studies of “risk-group” populations.

Vaccination is the most effective tool for preventing the importation of infectious disease pathogens [32]. In the Russian Federation, immunization against polio is regulated for children under 15 years of age arriving from countries endemic or at risk for polio, from migrant families or nomadic population groups, who are not vaccinated or have no information about vaccinations, upon arrival to Russia or upon identification [17]. Immunization protects the child, but cannot prevent virus excretion. The period of time between entry and detection can be quite long, and a potentially dangerous virus may be detected late or not detected at all. The introduction of mandatory vaccination certificates against polio is inconsistent with the existence of a visa-free regime with many countries and, unfortunately, does not exclude the possibility of falsification. Given this, preventive measures against the importation of poliovirus may include both immunization and timely organized virological investigation. The organizational aspects are probably the most difficult. They require intra-country and international cooperation, and ethical issues must be taken into account to avoid the stigmatization of the populations being surveyed.

The continued possibility of wPV1 or VDPV spread from still-existing foci of polio requires coordinated and timely action by polio-free countries to prevent the importation of the virus. Virological screening of children from “risk groups” can be an effective tool to achieve this goal, despite certain difficulties and limitations.

## Figures and Tables

**Table 1:** Isolation of viruses from “risk-group” children.

Year	No of Children Investigated			No. of Isolates											
	Total	Positive		PV Total		PV1		PV2		PV3		Mix		Non-PVs	
		No	%	No	%	No	%	No	%	No	%	No	%	No	%
2014	1231	71	5.8	13	18.3	1	7.7	4	30.8	5	38.5	3	23.1	58	81.7
2015	1099	54	4.9	14	25.9	2	14.3	2	14.3	8	57.1	2	14.3	40	74.1
2016	1127	63	5.6	19	30.2	0	0	7	36.8	12	63.2	0	0	44	69.8
2017	1071	52	4.9	7	13.5	6	85.7	0	0	0	0	1	14.3	45	86.5
2018	1036	47	4.5	3	6.4	0	0	0	0	3	100.0	0	0	44	93.6
2019	1202	83	6.9	15	18.1	8	53.3	0	0	6	40.0	1	6.7	68	81.9
2020	1067	15	1.4	4	26.7	0	0	0	0	4	100.0	0	0	11	73.3
2021	14,094	712	5.1	205	28.8	28	13.7	109	53.2	53	25.9	15	7.3	507	71.2
2022	23,425	667	2.8	134	20.1	33	24.6	0	0	77	57.5	24	17.9	533	79.9
2023	6196	313	5.1	18	5.8	3	16.7	0	0	12	66.7	3	16.7	295	94.2
Total	51,548	2077	4.0	432	20.8	81	18.8	122	28.2	180	41.7	49	11.3	1.645	79.2

**Table 2:** Epidemiologically significant PVs isolated from children of risk group in the Russian Federation in 2014–2023.

Virus	Source/ Gender/Age, Years	Child Country/ Region of Origin	Vaccination Status	Immunity Status	Place and Time of Detection	% nt sub/VP1	Reference
VDPV2	Healthy/m/1	Russia, Chechen Republic	Not vaccinated	Normal	Moscow, September 2016	1.12	[28]
VDPV2	Healthy/f/1	Russia, permanent residence Chechen Republic	Not vaccinated	Transient immuno-deficiency	Chechen Republic, December 2016	1.33	[28]
cVDPV2	Healthy/f/3	Tajikistan	1 bOPV	Normal	Voronezh, September 2021	4.32	[24]
cVDPV2	Healthy/f/3	Tajikistan	2 IPV	Normal	Moscow region, October 2021	3.10	[24]
Sabin-like PV2	Healthy/m/0.75	Egypt	3 OPV, 1 IPV	Normal	Zabaikalsky Krai, June 2021	0.33	–

VDPV—vaccine-derived poliovirus; cVDPV—circulating VDPV; OPV—oral poliovirus vaccine; bOPV—bivalent OPV; IPV—inactivated poliovirus vaccine.

**Table 3:** Cytopathogenic NPEVs isolated from children of risk group in the Russian Federation in 2014–2023.

*E. alphacoxsakie* *n* = 107		*E. betacoxsakie* *n* = 428		*E. coxsackiepol* *n* = 45	
Type	*n*	Type	*n*	Type	*n*
CVA4	45	CVB1–6	206	CVA19	12
CVA10	20	E11	52	CVA24	12
CVA2	11	E6	44	CVA1	6
CVA7	9	E13	29	CVA11	4
EV-A71	7	E7	22	CVA20	4
CVA5	6	E14	11	EV-C99	3
CVA3	2	E25	9	CVA13	2
CVA6	2	E3	7	EV-C96	2
CVA16	2	E18	6		
EV-A90	2	E29	6		
CVA14	1	CVA9	6		
		E30	5		
		E4	3		
		E12	3		
		E17	3		
		E21	3		
		E9	2		
		E19	2		
		E20	2		
		EV-B75	2		
		E1	1		
		E24	1		
		E26	1		
		E31	1		
		EV-B83	1		

## Data Availability

The data presented in this study are available in this manuscript.

## Abbreviations

The following abbreviations are used in this manuscript:

Polio	Poliomyelitis
WHO	World Health Organization
GPEI	Global Polio Eradication Initiative
PV	Poliovirus
wPV	Wild poliovirus
VDPV	Vaccine-derived poliovirus
Circulating VDPV	cVDPV
ITD	Intratypic differentiation
NPEV	Non-polio enterovirus
HAdV	Human adenovirus
AFP	Acute flaccid paralysis
OPV	Oral poliovirus vaccine
tOPV	Trivalent oral poliovirus vaccine
bOPV	Bivalent oral poliovirus vaccine
IPV	Inactivated poliovirus vaccine
nOPV2	New oral poliovirus vaccine type 2
